# Experimental Study on Primary Bird Co-Infection with Two *Plasmodium relictum* Lineages—pSGS1 and pGRW11

**DOI:** 10.3390/ani12151879

**Published:** 2022-07-22

**Authors:** Vaidas Palinauskas, Rita Žiegytė, Jakov Šengaut, Rasa Bernotienė

**Affiliations:** 1 Institute of Ecology, Nature Research Centre, Akademijos 2, 08412 Vilnius, Lithuania; rita.ziegyte@gamtc.lt (R.Ž.); dr@vetmed.lt (J.Š.); rasa.bernotiene@gamtc.lt (R.B.); 2 Faculty of Agrotechnologies, Vilnius University of Applied Sciences, 08303 Vilnius, Lithuania

**Keywords:** *Plasmodium relictum*, avian malaria, experiment, canary, multiple infection

## Abstract

**Simple Summary:**

Co-infections with several parasites in one host at the same time are common in the wild. It is conceivable that a host infected with several different parasites may be significantly more affected immunologically than a host infected with only one parasite. However, one plus one is not always two when talking about parasite infections. We followed the infection and some health parameters of twenty-four domestic canaries allocated to four groups: birds in two groups were infected with a single parasite (*Plasmodium relictum* genetic lineage pSGS1 or *P. relictum* pGRW11), in one group with both mentioned parasites, and birds in the fourth group were considered as the controls. After infection, the time of appearance of parasites in the blood and developed parasitemia did not differ between groups. Differences in hemoglobin levels and in the numbers of young red blood cells between the control and experimental groups were detected, but observed differences between the groups of infected birds were not statistically significant. This study shows that co-infection with two malarial parasites does not necessarily result in a greater number of parasites and more severe disease to the host.

**Abstract:**

Background: Co-infections are common in the wild. Thus, studies focused on parasite interactions are essential. We aimed to (i) follow the development of two genetic lineages of *Plasmodium relictum*—pSGS1 and pGRW11—during single infections and co-infections and (ii) evaluate their impact on bird host health. Materials: Twenty-four domestic canaries were allocated to four groups: two groups were infected with parasites of a single genetic lineage, one group was infected with parasites of both genetic lineages, and one group was considered as the control group. Parasitemia, the number of polychromatophils, changes in body weight, and hemoglobin levels were all quantified up to 32 days post-infection. Results: Three birds infected with pSGS1 died within 20 days post-infection. The prepatent period and the peak of parasitemia did not differ significantly between groups. Differences in hemoglobin levels between the control and experimental groups were observed and there was an abnormal increase in the number of polychromatophils in infected birds. In all infected groups, correlations were detected between the number of polychromatophils and parasitemia (positive), and between the number of polychromatophils and hemoglobin levels (negative). Conclusion: This study shows that co-infection with two phylogenetically closely related *P. relictum* parasites does not alter overall parasitemia and does not cause higher virulence to the host.

## 1. Introduction

Living organisms are infected with a multitude of parasites, with these pathogens commonly interacting with each other and their hosts. It is difficult or near impossible to elucidate the processes that occur during co-infections without experimental studies, and it is for this reason co-infection research is scarce. Nonetheless, such studies are extremely important given that the results obtained from studies based on the development and effects of single parasite infections on vertebrate hosts may not in fact reflect reality, particularly when the host is simultaneously infected with several parasites [1]. Co-infections are common in the wild. Thus, research untangling the interactions between several parasites, especially those occupying the same ecological niche, sharing development peculiarities, and similarly impacting host fitness, is crucial to better understand changes in parasite distribution, transmission, and further co-evolution [2]. Available data suggest that parasite interactions and their effects on the host vary widely depending on the type of parasites, the status of the host immune system, and other factors [1,2,3]. Malaria-causing parasites infect host red blood cells (RBCs) and interactions with any other parasites occupying this ecological niche may be important for the development and virulence of that malarial parasite. Co-infections with *Plasmodium* pathogens are very common in mammals and birds [4,5,6,7,8,9]. Therefore, studies dedicated to the interaction of these parasitic organisms, especially on species that are frequently found in the wild, are essential.

*Plasmodium relictum* is a species of avian malaria described more than 100 years ago [10]. This parasite is widespread and infects birds belonging to more than 300 different species [11]. In recent years, *P. relictum* has been listed as one of the most important invasive species [12]. After the development of molecular markers based on the mitochondrial cytochrome b (cyt b) gene, a vast diversity of avian malaria has been revealed [13]. After detailed studies on parasite morphologies of different genetic lineages (haplotypes of mtDNA cyt b gene fragment), researchers discovered that some closely related genetic lineages share the same morphology and can be considered the same species [14,15]. One of the best examples is the *P. relictum* clade, with its closely related lineages only differing by 0.2–3% on the mitochondrial cyt b gene and sharing an identical morphology; these are pSGS1, pGRW11, pGRW4, pLZFUS1, and pPHICOL01 [11,14,15,16,17]. Parasites belonging to some of these genetic lineages are often found in co-infections with each other and with parasites of other *Plasmodium* species ([6]; VP unpublished data). The development of *P. relictum* has mostly been studied under single infection experiments; however, its development under co-infection with different genetic lineages is comparatively rare [18,19,20,21,22,23,24,25,26,27,28,29]. By interacting with other malarial parasites, research suggests that *P. relictum* may have an effect on the development of other parasites, host health, and disease severity [18,21,23]. For instance, when co-infected with *P. elongatum*—an avian malarial parasite which infects both young (polychromatic) RBCs (pRBCs) and mature RBCs (mRBCs)—*P. relictum* (genetic lineage pSGS1) enhances the intensity of parasitemia of *P. elongatum* [18]. However, and counterintuitively, the development of *P. relictum* remains unaffected by this interaction. Another experimental study showed that *P. relictum* (lineage pSGS1) suppresses the development of the blood stages of *P. relictum* (lineage pGRW4) to the level that the parasite was undetectable in the blood [21]. Such contrasting effects of co-infections and the causes of the interactions between *P. relictum* pSGS1 and other malarial parasites are not entirely clear. More experimental studies of co-infections with various malaria parasites are needed to elucidate the mechanisms of action of these pathogens and to predict possible effects on other closely and distantly related malarial parasites.

We aimed to (i) determine the development of the blood stages of two *P. relictum* genetic lineages during single infections and co-infections and (ii) evaluate the relative impact of single versus co-infections on host health. For the experimental infections we used the globally distributed *Plasmodium relictum* genetic lineages pSGS1 and pGRW11 [30,31,32,33] and domestic canaries (*Serinus canaria domestica*) as vertebrate hosts. We experimentally demonstrated that the development of co-infection may not cause higher virulence altered by a more pathogenic parasite lineage, which is often observed in co-infection studies. We also encourage the international community to initiate discussions about the development of avian malarial lines and clones for future experimental research, which may facilitate the disentanglement of the processes and factors that are responsible for changes in disease development and epidemiology.

## 2. Materials and Methods

### 2.1. Ethical Statements

The procedures of the experimental infection of domesticated canaries were in accordance with the Directive of the European Parliament and the Council on the protection of animals used for scientific purposes and current laws of Lithuania. The permit was issued by the Ethical Committee for the use of Experimental Animals at the State Food and Veterinary Service of Lithuania (Ref. No. 2015/05/27-G2-27).

### 2.2. Obtained Parasites

We used *Plasmodium (Haemamoeba) relictum* parasites belonging to two genetic lineages: lineage pSGS1 (GenBank accession number JX993045) and lineage pGRW11 (JX993047). *Plasmodium relictum* parasite lineages pSGS1 and pGRW11 were isolated from two wild caught, naturally infected house sparrows (*Passer domesticus*) in June 2014 and 2011, respectively. Donor birds were caught at the Biological Station (Zoological Institute of the Russian Academy of Science) on the Curonian Spit in the Baltic Sea. The lineage of parasite infection in each donor bird was determined to be single using PCR-based methods and microscopy. Parasite lineages were cryopreserved in a biobank as described by [6] and later used for experimental procedures.

### 2.3. Experimental Procedures

The experiment was carried out in the vivarium of the Nature Research Centre (Vilnius, Lithuania) in 2017. Domestic canaries were commercially purchased and used as experimental animals. The birds were maintained in separate cages within a vector-free room under controlled conditions: 11:13 h light/dark photoperiod and 21 ± 1 °C air temperature. The birds were provided with food and water ad libitum. Based on the analysis of blood samples (microscopy and PCR, described below), all canaries were determined to be uninfected with malarial parasites prior to experimental procedures.

Twenty-four canaries were randomly allocated to four experimental groups (six birds in each group). One bird group (SGS) was infected with a single *P. relictum* pSGS1 lineage, the second group (GRW) was infected with a single *P. relictum* pGRW11 lineage, and the third group (MIX) was simultaneously infected with *P. relictum* of both pSGS1 and pGRW11 genetic lineages at a ratio of 1:1. The blood was taken from two donor birds at the same time and mixed in one tube. The fourth group (CON) received uninfected blood and was considered as the control group. Using a freshly prepared blood mixture (0.1 mL), the pectoral muscle of each bird was inoculated according to [22]. Prior to experimental infection, pSGS1 and pGRW11 isolates had been passed three and four times between birds, respectively. All infected birds from groups SGS, GRW, and MIX received a total of 3 × 10^5^, 6 × 10^5^, and 4 × 10^5^ RBCs with *P. relictum* meronts, respectively. Intensity of parasitemia prior to preparing blood samples for inoculation was quantified using microscopic examination of blood smears as described in Section 2.4.

Following inoculation, experimental birds were monitored every four days and up to 32 days post infection (dpi). The birds were weighed using an analytical balance (Kern & Sohn GmbH, Balingen, Germany). Peripheral blood (approximately 60 μL) was taken from all recipient birds after puncturing the brachial vein with a heparinized capillary. About 5 μL of peripheral blood was used for the preparation of two blood smears [34], 25 μL of peripheral blood was fixed in SET buffer (0.05 M Tris, 0.15 M NaCl, 0.5 EDTA, pH 8.0) for molecular detection and identification of the parasite [35], and the remaining peripheral blood (about 30 μL) was used to measure the amount of hemoglobin (Hb) using a HemoCue Hb201 System (Sweden). At the end of the experiment (32 dpi) all animals were euthanized by decapitation.

### 2.4. Microscopy of Blood Smears and Molecular Analysis

An Olympus BX51 light microscope (Olympus, Shinjuku City, Japan) was used to analyze blood smears. Parasitemia was estimated as a percentage by counting the number of infected RBCs per 10,000 RBCs [36]. To define the index of RBCs regenerative capacity in experimental and control birds, the percentage of immature RBCs (polychromatophils, pRBCs) was counted [18].

Total DNA from the blood stored in the SET buffer was extracted following an ammonium acetate DNA extraction protocol [37]. For the detection of *Plasmodium* DNA, a fragment of 479 bp of the parasite’s cyt b gene fragment was amplified using a nested PCR protocol [35]. We used the initial primers HaemNFI and HaemNR3 and nested primers HAEMF and HAEMR2 [38], which are specific to *Plasmodium* and *Haemoproteus* parasites. Thermal conditions followed [35]. One positive control (extracted DNA from bird blood microscopically positive for the *P. relictum*) and a negative control (ultrapure water instead of extracted DNA) were used every 12 samples. All reactions were carried out in 25 µL total volume, including 50 ng of total genomic DNA (2 µL), 12.5 µL of DreamTaq Master Mix (Thermo Fisher Scientific, Vilnius, Lithuania), 8.5 µL of ultrapure water, and 1 µL of each primer. Polymerase chain reaction products were evaluated on a 2% agarose gel and were sequenced with the primer HAEMF with an ABI PRISM TM 3100 (Applied Biosystems, Waltham, MA, USA). Sequences were edited using BioEdit 7.2 software (Raleigh, SC, USA) [39] and compared to sequences available on GenBank using the BLAST alignment tool (Available online: http//www.ncbi.nlm.nih.gov/BLAST (accessed on 10 December 2017)).

### 2.5. Statistics

The Statistica 7 software (StatSoft Inc., Tulsa, OK, USA) package was used to carry out statistical calculations. A non-parametric Mann–Whitney U test was used to compare non-independent values of the prepatent periods, parasitemias, and the numbers of certain blood cells on specific dpi between two experimental groups. A non-parametric Wilcoxon test was used to compare values of parasitemias, Hb, and pRBCs during the experiment between each experimental group. A Friedman test for repeated measures was applied to follow the changes in the investigated parameters within groups during the experiment. Spearman’s rank correlation coefficient was applied to assess the relationships between the investigated parameters. A *p*-value less than or equal to 0.05 was deemed to indicate statistical significance.

## 3. Results

### 3.1. Development of Parasites within Experimentally Infected Canaries

**Figure 1:**
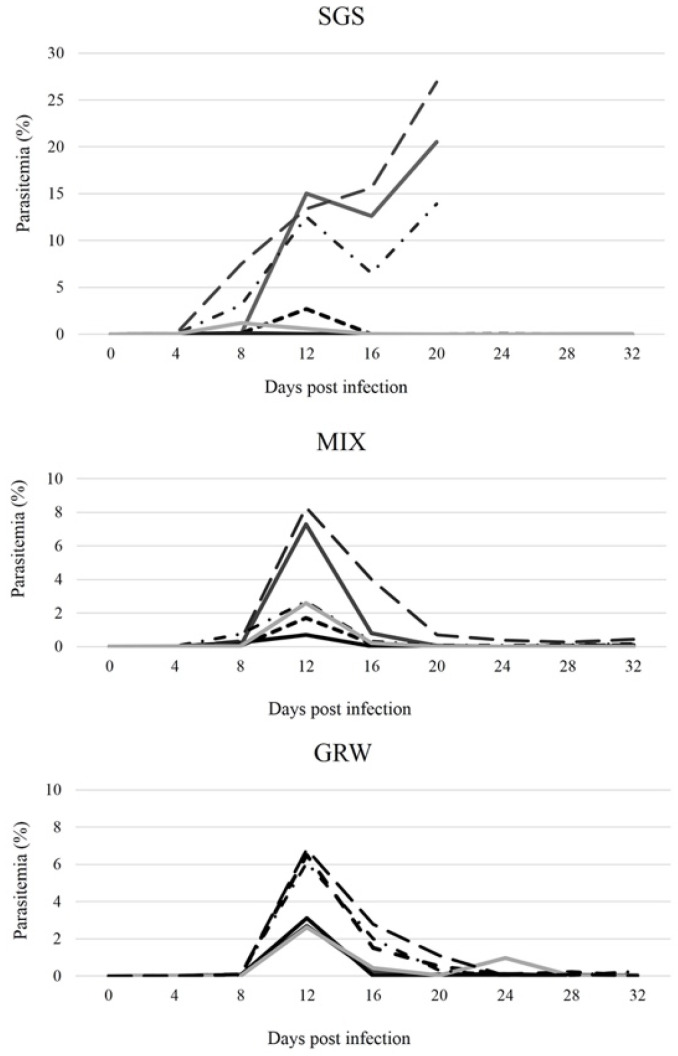
Dynamics of individual intensity of parasitemia (percentage of infected red blood cells) of experimentally infected canaries. SGS—birds infected with *Plasmodium relictum* (lineage pSGS1), GRW—birds infected with *P. relictum* (lineage pGRW11), MIX—birds co-infected with *P. relictum* pSGS1 and pGRW11.

Based on microscopic examination and PCR, all birds from the CON group remained uninfected throughout the experiment. Infections developed in all birds from the SGS, GRW, and MIX groups, and all birds remained infected for the entire duration of the experiment. For most of the birds, the prepatent period—the time between inoculation with the parasite and the appearance of blood stages—was within 4 dpi. However, for one bird from the MIX group and two birds from the GRW group, the prepatent period was within 8 dpi. The prepatent period did not differ significantly between groups (SGS/GRW, z = 0.88, *p* = 0.4, df = 10; SGS/MIX, z = 0.40, *p* = 0.7, df = 10). All birds from the CON, GRW, and MIX groups survived until the end of the experiment (32 dpi). However, three birds from the SGS group died; the survival of the SGS group was on average 26 ± 5.6 dpi. The three birds from the SGS group died 20 dpi when the pSGS1 infection peaked at 13.9%, 20.5%, and 26.9% intensities, respectively. These intensities were the highest out of all the experimentally infected birds (Figure 1).

In the infected birds from the GRW and MIX groups, peak parasitemia was observed 12 dpi (Figure 1). In the SGS group, peak parasitemia of 5 out of 6 birds was reached at 8 and 12 dpi. Following these peaks, parasitemia began declining for most of the birds. However, in the case of the SGS group, a second, sharp increase in parasitemias was observed which led to the death of three birds 20 dpi. Despite the high parasitemias in the SGS birds (on average 10.9 ± 9.5% between 8 and 20 dpi), peak parasitemias in the SGS group did not significantly differ compared to the birds in the GRW (on average 4.6 ± 1.7%, 12 dpi, df = 10, z = 0.66, *p* = 0.51) or MIX groups (on average 3.9 ± 2.7%, 12 dpi, df = 10, z = 0.64, *p* = 0.52). As a general pattern, *P. relictum* parasitemias decreased to chronic levels (<0.7%) 20 dpi in all surviving experimental birds (Figure 1). Three birds from the SGS group developed high (>10%)parasitemias and all three birds subsequently died 20 dpi (Figure 1).

### 3.2. Changes of Health Parameters

**Figure 2:**
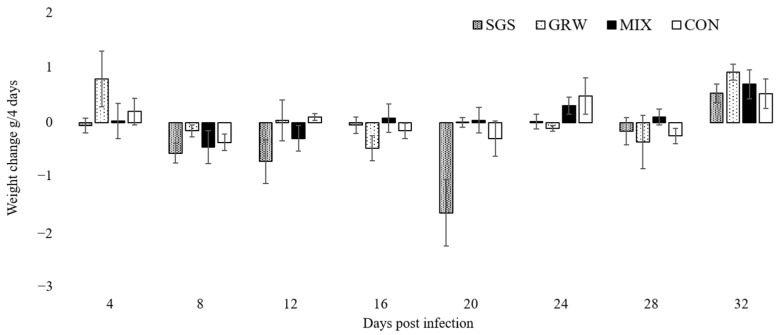
Body weight changes of experimentally infected and control canaries. SGS—birds infected with *Plasmodium relictum* (lineage pSGS1), GRW—birds infected with *P. relictum* (lineage pGRW11), MIX– birds co-infected with *P. relictum* pSGS1 and pGRW11, CON—control birds. Error bars represent standard errors.

A decrease of weight was only observed in birds from the SGS group up to 20 dpi (χ^2^ =11.73, *p* = 0.02), whereas the weights of the birds in the other two experimental groups (GRW, *p* = 0.08; MIX, *p* = 0.2) and the CON group (*p* = 0.1) remained stable (Figure 2).

**Figure 3:**
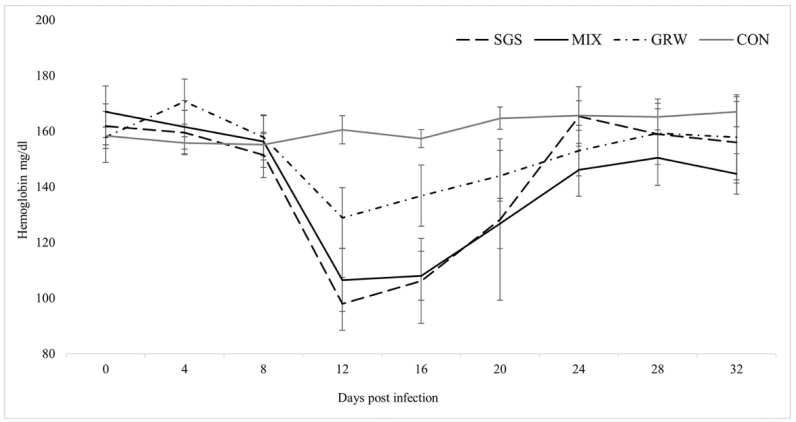
Values of mean hemoglobin level in experimentally infected and control birds. Symbols are as in Figure 2.

Changes in the Hb level of the experimental birds during the experiment were found in all experimental groups (SGS, χ^2^ = 14.96, *p* = 0.04; GRW, χ^2^ = 23.84, *p* = 0.00; MIX, χ^2^ = 32.57, *p* = 0.00). No significant differences were detected between the experimental groups (SGS/MIX, z = 0.24, *p* = 0.8; GRW/MIX, z = 1.8, *p* = 0.07). Significant differences in the Hb level between the CON and all the other groups except the GRW group were detected (SGS, z = 2.01, *p* = 0.04; GRW, z = 1.84, *p* = 0.07; MIX, z = 1.96, *p* = 0.05). This difference was detected at 12 dpi (SGS, z = 2.8, *p* = 0.005; MIX, z = 2.8, *p* = 0.005), at 16 dpi (SGS, z = 2.3, *p* = 0.02; MIX, z = 2.8, *p* = 0.005), and at 20 dpi (MIX, z = 2.5, *p* = 0.01) (Figure 3). A significant correlation between the Hb level and the parasitemia (R2 = −0.72, *p* = 0.00) was detected only in the SGS group.

**Figure 4:**
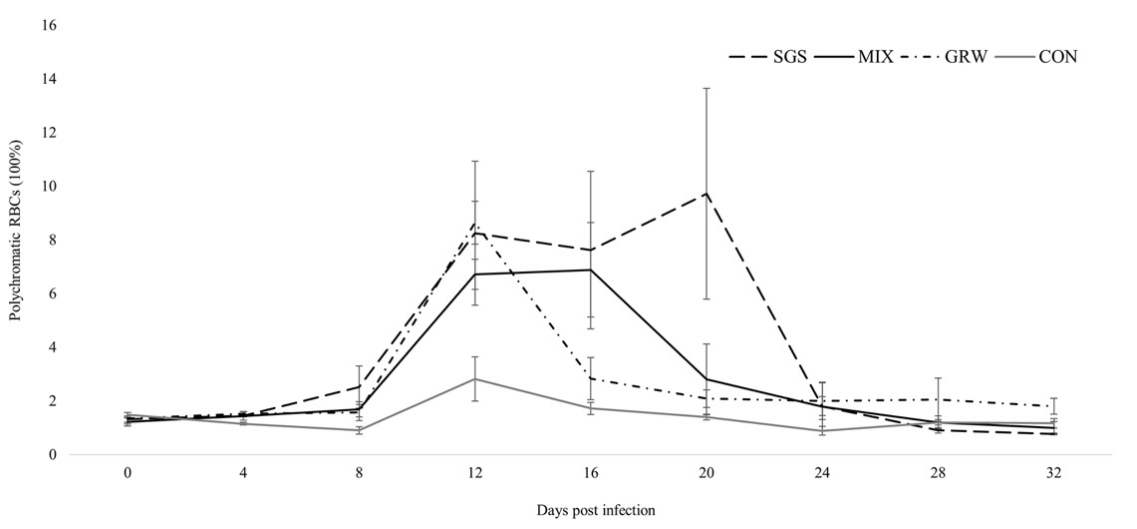
Percentage of polychromatic (i.e., young) red blood cells in experimentally infected and control birds. Symbols are as in Figure 2.

Polychromatophilia—an abnormal significant increase in the number of pRBCs—was observed in all of the experimentally infected birds (Figure 4) (GRW, z = 2.55, *p* = 0.01; MIX, z = 2.10, *p* = 0.04), but this increase was not significant in the SGS group (z = 1.72, *p* = 0.09). Compared to the CON group, experimentally infected birds experienced significant increases in pRBCs 12 dpi (GRW, z = 2.6, *p* = 0.00; MIX, z = 2.6, *p* = 0.00), 16 dpi (MIX, z = 2.7, *p* = 0.00), and 20 dpi (SGS, z = 2.2, *p* = 0.03). No significant differences were detected between the birds with mixed and single infections, or between the birds with different single infections (SGS/MIX groups, z = 1.2, *p* > 0.05; GRW/MIX groups, z = 0.8, *p* > 0.05; SGS/GRW groups, z = 0.5, *p* > 0.05). A positive correlation was observed between parasitemia and the number of pRBCs in the SGS, GRW, and MIX groups (SGS, R2 = 0.77, *p* = 0.00; MIX, R2 = 0.41, *p* = 0.00; GRW, R2 = 0.74, *p* = 0.00). A negative correlation was observed between the number of pRBCs and the amount of Hb in the experimentally infected birds from all of the experimental groups (SGS, R2 = −0.66, *p* = 0.00; GRW, R2 = −0.35, *p* = 0.01; MIX, R2 = −0.57, *p* = 0.00). There was no correlation between the number of pRBCs and the amount of hemoglobin in the birds from CON group (R2 = 0.05, *p* > 0.05).

## 4. Discussion

In this experimental study, we show the development of blood stages of two *P. relictum* genetic lineages in a single and co-infection arrangement. We also compare single and co-infection impacts on host health parameters and discuss the importance of determining different isolates of the same genetic lineage.

The birds from all experimental groups were susceptible to infections and the time of appearance of the infection in the blood did not differ between the groups. In a study by Palinauskas et al. [18], the authors did not find significant differences in the prepatent period of birds infected with a single pSGS1 lineage and those co-infected with pSGS1 and *P. elongatum* pERIRUB01 lineages. However, the appearance of pERIRUB01 in the blood was altered by the presence of the pSGS1 lineage. These two parasite lineages have preferences for different types of erythrocytes. Thus, the early appearance of pSGS1 in the blood activated the erythropoietic system, resulting in increased numbers of pRBCs preferred by *P. elongatum* and in turn leading to a shorter prepatent period. In this study, the genetic lineages were closely related, with both lineages showing the same preference for mRBCs only. Thus, the increased number of pRBCs in the blood did not affect the development of the parasites of any genetic lineage. In another study where siskins and crossbills were infected with the *P. relictum* pSGS1 lineage and the *P. ashfordi* pGRW2 lineage, contradictory results were obtained [23]. The prepatent period of the pSGS1 lineage did not differ between crossbills infected with a single pSGS1 lineage and those co-infected with the pGRW2 lineage. However, in siskins, the prepatent period of the pSGS1 lineage during co-infections was shorter compared to those with a single infection. Thus, our results suggest that the level or changes to the prepatent period of infection may depend not only on a combination of parasite lineages, but also on the host species.

After appearance in the blood, the parasitemia developed in all of the experimentally infected birds (Figure 1). A bell-shaped developmental curve, which is typical for *P. relictum* [10,20,22,25,26,29], was observed during co-infection as well. The peaks of parasitemia varied between individuals from all three of the experimental groups. Although in some SGS birds the peaks of parasitemia reached over 20%, the mean peak in the SGS group did not differ significantly from that of the GRW or MIX groups and likely resulted from the small experimental groups and large data scattering in the SGS group. In all of the experimental groups, parasitemias peaked 12 dpi and thereafter declined. However, three birds infected with a single pSGS1 lineage were not able to cope with the parasitemia, which, after a slight decline, started sharply increasing 16 dpi, eventually leading to the death of these infected birds. Severe anemia is the most probable cause of death. Exo-erythrocytic stages of the parasite (i.e., phanerozoites) may also cause lethal disease and death of the host [40], but this might be not the case for these parasites. According to older literature, *P. relictum* phanerozoites develop in different internal organs and should be detectable in infected birds [10,41]. However, according to more recent studies on *P. relictum* parasites, these stages were not observed in different internal organs in the birds infected with different lineages of *P. relictum* ([15]; MI pers. comm.). Therefore, experimental studies and detailed descriptions of the exo-erythrocytic stages of *P. relictum* parasites are needed.

Previous studies revealed that the intensity of parasitemia of one avian malarial parasite can be enhanced by another *Plasmodium* pathogen during co-infection [18]. However, in this study, we were not able to untangle the development peculiarities of the blood stages of each parasite in the blood of co-infected birds. This is because of the morphological and genetic similarity of the studied parasite lineages. In a study by Aželytė et al. [21], analyses of the development of two *P. relictum* lineages, pSGS1 and pGRW4, during co-infection were obtained by designing lineage-specific primers and applying quantitative PCR methods. However, the attempts to design specific primers for pSGS1 and pGRW11 were not successful as these two genetic lineages were too similar, with studies reporting recombination events [42] and evidence of hybridization [43]. However, the overall parasitemia of infection in the MIX group did not differ significantly from any of the single infections and did not show increases of parasitemia caused by the interaction of these two parasite lineages. The ecological niche of these pathogens clearly overlaps, with both parasite lineages specializing in and infecting only mRBCs. This could cause direct competition between parasites. Antagonistic interaction between specializations for mRBCs was demonstrated in another experimental study, where the increase of one parasite lineage caused a decrease in parasitemia of another [23]. An example of such an antagonistic interaction causing the decrease of one parasite lineage during co-infection was illustrated by the simultaneous experimental infection of birds with *P. relictum* pSGS1 and pGRW4 parasite lineages, where the presence of the pSGS1 lineage suppressed the development of the pGRW4 lineage [21]. Other examples of co-infections with different specializations of parasites show that the presence of one parasite may enhance the intensity of parasitemia of another parasite due to co-infection [18]. These differences may be caused by factors such as the limitation of the ecological niche or rather by more complicated interactions between the two parasites or between the parasite and the host. Several studies demonstrated that malarial parasites with preferences for only young or young and mature erythrocytes have an advantage during co-infections [7,8,18], while parasites infecting only mRBCs compete with each other and/or do not have a competitive advantage during co-infection ([18,23]; present study).

Virulence expressed by measures of mortality, anemia, weight loss, and changes in Hb levels were quantified from the experimental and control birds. Three out of six birds infected with a single pSGS1 lineage died during experimentation, while all birds simultaneously infected with the pSGS1 and pGRW11 lineages survived for the duration of the experiment. This illustrates that co-infection may be less virulent when compared to single infection with a more virulent parasite. Experimental studies with mixed infections usually reveal higher virulence of infection due to the interaction of two pathogens. One malarial parasite may facilitate the development of another malarial parasite in infected hosts, which may lead to higher virulence. A recent experimental study with canaries showed that the virulence of co-infection with *P. relictum* pSGS1 and *P. elongatum* pERIRUB01 was similar to a single highly virulent *P. elongatum* parasite [18], but the interaction of the two parasites caused different pathologies. For example, birds infected with a single *P. elongatum* lineage had non-regenerative anemia, while lethal regenerative anemia was observed in birds co-infected with both *P. relictum* pSGS1 and *P. elongatum* pERIRUB01 lineages. In our study, co-infection did not show higher virulence in birds with both pSGS1 and pGRW11 lineages. Compared to the CON group, the decrease of weight was only recorded in birds infected with a single pSGS1 lineage up to 20 dpi. The decline in weight within the SGS group was driven by the three birds which developed the highest peaks of parasitemia and died within 20 dpi (Figure 2). During periods of high parasitemia between 16 and 20 dpi, the weight of the surviving birds decreased by 0.4 g ± 0.01 within 4 days, while the weight of the three birds highly infected with parasites before their death decreased on average by 2.9 g ± 0.7. The evaluation of weight is not sensitive during lighter infections, especially when the food is provided ad libitum [21,22,23,25]. However, if these pathogens cause lethal disease, there is a clear sign of weight loss several days before the death of the host [18,26]. Other health parameters such as the Hb level did not differ between birds with single and co-infection, showing that the effect on host health was the same in birds with single infections and co-infections. Similar results were recorded concerning the increase in the number of polychromatic erythrocytes and no differences between experimental groups were detected. There was a positive correlation between parasitemia and the number of pRBCs in all three of the experimental groups, a result substantiated by other studies [18].

Several experimental studies have used the same genetic lineage of avian malarial parasites, which showed differing virulence to the same bird species. In our study, the pSGS1 lineage of *P. relictum* obtained from naturally infected house sparrows caused 50% mortality in experimentally infected canaries. A recent experimental study showed that pSGS1 obtained from common crossbills caused 17% mortality in canaries [18]. It might be that some isolates of the same species and even genetic lineage may cause different disease. Early studies on avian malaria pointed out, for instance, *P. relictum* virulence differences between Italian or German strains, but a more precise evaluation or clarification of different isolates or clones was lacking. As avian malaria is useful for different experimental studies, both for a better understanding of avian malarial pathogens and for their use as model organisms to study general aspects of *Plasmodium* parasites and infectious diseases, we call for the international community to discuss the best ways of organizing the biobank with different clones of avian malaria pathogens.

## 5. Conclusions

In conclusion, this study shows that co-infection with two phylogenetically closely related *P. relictum* parasites belonging to the pSGS1 and pGRW11 genetic lineages does not alter overall parasitemia. This phenomenon may be caused by direct competition between the two parasites with the same specialization in mRBCs. The virulence of co-infection can be the same compared to infections with single parasite lineages, which could be related to the specific combination of lineages used. As evidence from experimental research revealed differences in virulence between different isolates of the same lineage, we call for studies of different isolates of avian malarial parasites and ask the international community to initiate a biobank database containing the variously developed clones of these pathogens, which could be used in future experimental studies.

## Data Availability

The data supporting the reported results are available upon email inquiry.
